# Personality and unachieved treatment goals related to poor adherence to asthma medication in a newly developed adherence questionnaire – a population-based study

**DOI:** 10.1186/s40248-016-0078-8

**Published:** 2016-12-05

**Authors:** Malin Axelsson, Linda Ekerljung, Bo Lundbäck, Jan Lötvall

**Affiliations:** 1 Krefting Research Centre, Institute of Medicine, Internal Medicine and Clinical Nutrition, Sahlgrenska Academy, University of Gothenburg, Box 424, SE-405 30 Gothenburg, Sweden; 2 Department of Care Science, Faculty of Health and Society, Malmö University, Jan Waldenströms gata 25, SE-205 06 Malmö, Sweden

**Keywords:** Medication adherence, Patient compliance, Population-based study, Respiratory tract disease, Self-report

## Abstract

**Background:**

Health-care professionals have a responsibility to be attentive to patients’ adherence behavior but it could be difficult to identify poor adherence in the context of clinical practice. Assessment of personality could be used to identify individuals who are in need for support with their adherence behavior. To our knowledge, existing adherence questionnaires are not based on individuals reflecting asthmatics in the general population and there is limited research describing adherence with asthma medication in relation to personal goals with the treatment. The aim was to develop and validate an adherence questionnaire in adult individuals with asthma from the general population and to assess adherence in relation to personality traits and goals with the asthma medication using the developed questionnaire.

**Methods:**

The study was conducted in three phases: 1. A preliminary postal 46-item questionnaire was refined after psychometric testing (*n* = 157). 2. The questionnaire was validated (*n* = 104). 3. The developed adherence questionnaire was analyzed in relation to personality traits and achieved goals with the asthma medication. Adult respondents with physician diagnosed asthma using asthma medications were selected from the population-based West Sweden Asthma Study. The respondents completed the Neuroticism, Extraversion and Openness to Experience Five-Factor Inventory and the Medication Adherence Report Scale and stated their goals with the asthma medication. Data were analyzed using t-tests, correlations, multiple regression and principal component analysis.

**Results:**

A final questionnaire was developed consisting of ten items organized in three subscales - “medication routines”, “self-adjusting the medication” and “concerns about side-effects”. Two of the subscales - “medication routines” and “self-adjusting the medication” – were associated with the Medication Adherence Report Scale. The subscale “medication routines” was associated with the personality traits – Conscientiousness and Neuroticism and unachieved goals with the asthma medication.

**Conclusions:**

The developed questionnaire appears to be useful for measuring adherence to asthma medication in adult individuals with asthma. The study suggests that both individual differences and personal treatment goals need to be addressed in efforts to promote adherence to asthma medication treatment.

## Background

Several studies have reported that adherence to asthma medication treatment among individuals with asthma could be better [1–4]. At the same time, health-care professionals have a responsibility to promote improved adherence [5], and it has been recommended that adherence to asthma medication be checked during follow-ups at asthma clinics [6]. However, as regards adherence estimations in daily clinical practice, it may be difficult to estimate accurate adherence levels. One common method of estimating adherence to medication treatment is through self-report, which usually is conducted using questionnaires or interviews about medication use [7]. This method of estimating adherence has the advantage of being cost-effective and suitable for use in daily clinical practice [5]. Though, self-report as a method has drawbacks in terms of recall bias [8] and social desirability bias [5, 7], the latter possibly resulting in overestimation of adherence [5]. Comparing self-report to other methods used to assess adherence, one study showed positive associations between self-reported adherence and adherence measured electronically [9], but another study demonstrated that canister weight and electronic monitoring were more reliable than self-reports [10]. Still, a meta-analysis concluded that self-reported adherence gives a good estimation of adherence to medication treatment [11]. It has also been argued that self-reports of low adherence can be regarded as reliable [5]. Additionally, self-reports provide valuable information of patients’ problems with adherence. Canister weight and electronic monitoring do not provide that kind of information and these two adherence measurements may not be possible or suitable to use in daily clinical practice [7].

It has also been argued that it may be difficult to identify which patients are likely to deviate from a prescribed treatment [7]. Previous research has shown that assessment of personality traits could be one method to identify personal resources and needs of significance for adherence behavior in relation to asthma medication treatment [12–14]. The five-factor model describes personality in terms of five broad and bipolar personality traits: Neuroticism, Extraversion, Openness, Agreeableness and Conscientiousness [15]. In individuals with asthma, Neuroticism has been associated with lower adherence [14], while Conscientiousness has been associated with higher adherence to asthma medication treatment [13, 14].

Asthma control [12, 16] and health-related quality of life [17, 18] are often used as outcome measures of adherence to asthma medication. For instance, Horne et al. showed [16] that better symptom control was observed among individuals with asthma, who reported higher adherence to the medication treatment. Higher adherence has also been associated with better perceived health-related quality of life [17]. To the best of our knowledge, there is limited research describing adherence to asthma medication in relation to personal goals with the medication treatment.

In summary, adherence to asthma medication could be improved [1–4], and health-care professionals have a responsibility to check adherence [6] but it could be difficult to identify poor adherence in the context of clinical practice and to identify which patients are more likely to deviate from a prescribed treatment [7]. There is limited research describing adherence in relation to whether personal goals with the asthma medication have been achieved or not. To the best of our knowledge, existing adherence questionnaires are not based on individuals reflecting asthmatics in the general population.

## Methods

### Aim

The aim was first to develop and validate an adherence questionnaire in adult individuals with asthma from the general population and second to assess adherence in relation to personality traits and goals with the asthma medication using the developed questionnaire.

### Procedure

The study was conducted in three phases:

1. Development of the adherence questionnaire
2. Validation of the developed adherence questionnaire
3. Assessment of adherence in relation to personality traits and achieved goals using the developed questionnaire

#### Phase 1 – questionnaire development

An “item-pool” consisting of 46 items was generated based on literature on adherence in combination with our experience and knowledge from our previous research (Table 1) [12, 14, 19, 20]. A 46-item questionnaire was constructed that began by explaining the aim of the questionnaire in an accepting tone. In order to reduce the risk of social desirability bias, it has been recommended that questions about adherence be posed in a way that presents missed doses as something normally occurring [21]. The respondent was asked to take a position on each statement by choosing among five response alternatives (“Doesn’t correspond at all”, “Doesn’t correspond very well”,”Neither corresponds nor doesn’t correspond”, “Corresponds fairly well”, and “Corresponds exactly”) on a 5-point Likert-type scale [22]. The order of the items was constructed so that the questionnaire did not begin with controversial or emotive items. There was also a mixture of positive and negative statements, which is suggested as a method of minimizing response bias [23].

**Table 1:** The item pool. Items marked 1 and 2 were used in psychometric tests in phase 1 and 2

	The condition i.e. asthma
	My asthma is mild.
	My asthma will never go away.
1	I’ve doubted at times that I really have asthma.
1	My asthma comes and goes.
	The treatment
	I’m not sure about my inhalation technique.
	My asthma inhalator is user friendly.
	I don’t know how my asthma medicine works.
	My asthma inhalator is difficult to use.
	I have different inhalators with asthma medicine and I don’t really know when I should use one or the other.
	The health care
	I want to participate in deciding on my asthma medication.
	I get enough information about my asthma medicine from my nurse/doctor.
	I wish I got more information about my asthma medicine.
	The individual – beliefs about asthma medication
	I avoid unnecessary asthma attacks by taking my medicine.
1, 2	I’m worried about how my asthma medicine will affect my body in the long run.
	My asthma medicine prevents me from feeling worse on account of my asthma.
	My asthma medicine is a source of security.
1, 2	I worry about the side-effects of asthma medicine.
1	Thanks to my asthma medicine I hardly know I have asthma.
	My asthma could get worse in the future if I don’t take my asthma medicine now.
1	I’m tired of taking my asthma medicine.
	I’m sceptical about my asthma medicine.
1	I don’t like taking medicine unless I feel I need it.
	My asthma medicine makes me feel safe and secure.
	I think the advantages of taking asthma medicine outweigh the disadvantages of not taking it.
	My asthma medicine makes me feel good and lets me manage my life well.
	The individual – personal characteristics
1	I don’t feel I’m participating as far as my asthma medicine is concerned.
	I prefer cooperating.
	I don't like discipline and routines.
	I’m a rather insecure person.
	I’m usually suspicious of other people’s intentions.
	To remember to take asthma medicine you have to have routines and I’m not really that kind of person. I’m a bit more unstructured.
	I often feel worried.
	I’m a meticulous person and have routines for my asthma medicine.
	The individual – adherence behavior
1, 2	It’s easy to forget asthma medicine.
1, 2	I don’t follow the doctor’s prescription exactly but instead I can feel what I need to take. ^2^I don’t follow the doctor’s prescription of asthma medicine exactly but instead I can feel how much I need to take.
	I really have to struggle to not forget to take my asthma medicine.
	Routines are important for helping me remember to take my asthma medicine.
1, 2	I don’t have any real habits for my asthma medicine but instead I take them when I remember to.
	I can’t manage if I don’t take more asthma medicine than is prescribed.
1, 2	I don’t use my asthma medicine exactly like my doctor has said.
1, 2	Sometimes I test going without my asthma medicine to see what it’s like.
1, 2	I don’t really follow the instructions for my asthma medicine but instead I can feel what my body needs. If I feel I need more, I take more. If I feel I need less, I take less.
1	I feel I need to take more reliever medicine than is prescribed.
1, 2	I can tell myself how I feel and how much of my asthma medicine I need to take.
1, 2	I’m like a periodical drinker as far as my asthma medicine is concerned.
	Even though it would be good if I had a routine for my asthma medicine it’s probably nothing I’m going to do anything about.

The questionnaire was sent by mail to 300 adult individuals with asthma using asthma medication, selected at random from the population-based West Sweden Asthma Study (WSAS). The following is a brief explanation of the WSAS. In 2008, 30,000 randomly selected individuals were invited to take part in the WSAS, by postal questionnaires, and 18,087 completed and returned the questionnaires [24]. Out of the 18,087 individuals from the general population who completed the questionnaires, 2006 individuals, including 964 adult asthmatics, participated in the following clinical phase of the WSAS . The respondents in the current study have been selected from the asthma cohort (*n* = 964), who reported that they had used asthma medication during the past 12 months. In 2013, they were invited to participate through one initial mailing of the questionnaire and one reminder. A completed and returned questionnaire was regarded as consent to participate in the current study.

#### Phase 2 – validation of the developed questionnaire

Following psychometric analysis of the questionnaire in phase 1, a new questionnaire was constructed, similar in design to that used in phase 1. However, one item was slightly changed from “I don’t follow the doctor’s prescription exactly but instead I can feel what I need to take” to “I don’t follow the doctor’s prescription of asthma medicine exactly but instead I can feel how much I need to take.” The change was made in order to clarify that the item was supposed to assed the self-adjusting of the doses, which was not clear in the origin item “what I need to take” and to emphasize that it was asthma medication that was in focus.

In 2013, 200 adult individuals with asthma using asthma medication were invited to take part in phase 2 of the current study i.e. the validating of the developed adherence questionnaire. They were randomly selected from the asthma cohort in the clinical phase of the WSAS. They had not been included in phase 1 described above. They received the adapted questionnaire and, in addition, the Medication Adherence Report Scale (MARS) [25]. One initial mailing of the questionnaire and one reminder were sent. A completed and returned questionnaire was regarded as consent to participate in the study.

#### Phase 3 - assessment of adherence in relation to personality traits and achieved goals using the developed questionnaire

The sample in phase 3 consisted of the same respondents as in phase 2 described above. The respondents completed the following questionnaires on personality traits and goals with the asthma medication:

In order to assess personality traits, the Neuroticism, Extraversion and Openness to Experience Five-Factor Inventory (NEO-FFI) consisting of 60 items, scaled 1–5, was used [15].

In order to assess goals with the asthma medication treatment the following two questions were used:

1. “What is your goal with your asthma medication?” This was an open question and the respondents stated their personal goals with the asthma medication.
2. “Do you think that your goal with your asthma medication has been reached?” This question was used to assess whether the stated goal with the asthma medication was reached or not. Yes and no were used as response alternatives.

#### Analyses – phases 1–3

The study samples were described using descriptive statistics, i.e. frequencies, percentages, means and standard deviations [26]. Factor analysis, i.e. principal component analysis with Varimax and Kaiser normalization as the rotation method, was used to explore associations between the items, to remove redundant items and to identify underlying constructs among the items [27]. Cronbach’s alpha was used to test the reliability of the scales in the developed adherence questionnaire [26]. In order to demonstrate validity, Pearson’s correlation coefficient was used to determine associations between responses to the new questionnaire and the MARS (in phase 2) [23]. Associations between adherence and personality traits were investigated using Pearson’s correlation coefficient and a multiple regression model. T-tests were used to study differences between subgroups. All statistical analyses were performed using SPSS version 20. Reports on goals with the asthma medication were compiled into categories according to content.

### Ethical considerations

The study was approved by the regional research ethics board at the University of Gothenburg reference number 926–12 and adhered to Declaration of Helsinki-Ethical Principles for Medical Research Involving Human Subjects [28].

## Results

### Phase 1 – questionnaire development

Out of the 300 individuals with asthma who were invited to participate, 157 returned completed questionnaires, resulting in a response-rate of 52 %. Regarding disease severity, 15 % of the 157 respondents had made an emergency visit due to asthma during the last 12 months. Additional characteristics of the respondents are presented in Table 2. It has been recommended that items with high/low endorsement should be avoided in questionnaires, as a skewed distribution leads to poor discriminatory power. For that reason, items were considered for removal if ≤ 20 % or ≥ 80 % of the responses endorsed either “Doesn’t correspond at all”/“Doesn’t correspond very well” or “Corresponds fairly well”/”Corresponds exactly”) [22]. Eventually, 29 items that did not show a good response spread were removed from further analysis. Table 1 shows the 17 items that were used in the subsequent psychometric analyses.

**Table 2:** Background characteristics of the respondents

	Study 1 (*n* = 157)	Study 2 (*n* = 104)
	Frequencies (%)	Frequencies (%)
Men	66 (42)	39 (38)
Women	91 (58)	65 (62)
Age mean (SD^a^)	50 (15)	49 (14)
Asthma inhalers		
Corticosteroids	64 (41)	38 (36)
Combination therapy (corticosteroids + long-acting beta^2^-agonists)	40 (25)	30 (29)
Short-acting beta^2^-agonists	84 (53)	64 (61)
Long-acting beta^2^-agonists	17 (10)	10 (9)

^a^standard deviation

Two factor analyses were conducted. In the first factor analysis with 17 items (Table 1), the Kaiser-Meyer-Olkin and Bertlett’s test showed a sampling adequacy of 0.766, approx. and a significance of <0.001. The analysis resulted in five components with an Eigenvalue >1, which were consistent with the generated scree plot. The variance was 62.67 %. The internal consistency of the five preliminary components was measured using Cronbach’s α. The consistency analysis showed that the three items with the lowest loadings (≤0.45 in the first two factors and <0.65 in the third factor) on each of the three first components also had the lowest corrected item-total correction and lowered the Cronbach’s α. The four items underlying the fourth and fifth component had a Cronbach’s α ≤ 0.7 and were discarded from further analyses. An α ≥ 0.7 is recommended for a questionnaire that is under development, while an α ≥ 0.8 is recommended for a more established one [23]. In total, seven items were removed before proceeding to the next analysis.

The second factor analysis was based on the ten remaining items (Table 1). The Kaiser-Meyer-Olkin and Bertlett’s test showed a sampling adequacy of 0.745, approx. and a significance of <0.001. The analysis resulted in three components with an Eigenvalue >1, which were consistent with the scree plot. The variance was 69.31 %. The three factors were named “medication routines”, “self-adjusting the medication” and “concerns about side-effects”; they were then subjected to reliability tests. The factor loadings and Cronbach’s α are presented in Table 3.

**Table 3:** Factor loadings from Principal component analysis with Varimax and Kaiser normalization as the rotation method for the final three-factor solution and Cronbach’s α in phase 1

	“medication routines”	“self-adjusting the medication”	“concerns about side-effects”
“medication routines” Cronbach’s α = 0.802			
It’s easy to forget asthma medicine.	0.842	-0.018	0.027
I don’t have any real habits for my asthma medicine but instead I take them when I remember to.	0.794	0.243	-0.089
I’m like a periodical drinker as far as my asthma medicine is concerned.	0.723	0.252	0.011
Sometimes I test going without my asthma medicine to see what it’s like.	0.627	0.303	0.085
I don’t use my asthma medicine exactly like my doctor has said.	0.500	0.458	0.305
“self-adjusting the medication” Cronbach’s α =0.769			
I don’t really follow the instructions for my asthma medicine but instead I can feel what my body needs. If I feel I need more, I take more. If I feel I need less, I take less.	0.182	0.900	0.047
I can tell myself how I feel and how much of my asthma medicine I need to take.	0.132	0.779	-0.188
I don’t follow the doctor’s prescription exactly but instead I can feel what I need to take.	0.275	0.698	0.086
“concerns about side-effects” Cronbach’s α = .922			
I’m worried about how my asthma medicine will affect my body in the long run.	0.009	-0.034	0.949
I worry about the side-effects of asthma medicine.	0.036	0.016	0.957

### Phase 2– validation of the questionnaire developed in phase 1 described above

Out of the 200 individuals with asthma who were invited to participate in the validating of the questionnaire, 104 returned completed questionnaires, resulting in a response rate of 52 %. Regarding disease severity, 16 % of the 104 respondents had made an emergency visit due to asthma during the last 12 months. Additional characteristics of the respondents are presented in Table 2.

In the factor analysis with the ten items selected from phase 1, the Kaiser-Meyer-Olkin and Bertlett’s test showed a sampling adequacy of 0.811, approx. and a significance <0.001. The analysis resulted in a three-factor solution with an Eigenvalue > 1, which was consistent with the scree plot. The variance was 79.91 %. The three components - “medication routines”, “self-adjusting the medication” and “medication concerns” - were then subjected to reliability tests. The factor loadings and Cronbach’s α are presented in Table 4.

**Table 4:** Factor loadings from Principal component analysis with Varimax and Kaiser normalization as the rotation method for the final three-factor solution and Cronbach’s α in phase 2

	“medication routines”	“self-adjusting the medication”	“concerns about side-effects”
“medication routines” Cronbach’s α = 0.880			
It’s easy to forget asthma medicine.	0.886	0.192	0.017
I’m like a periodical drinker as far as my asthma medicine is concerned.	0.829	0.340	-0.096
I don’t have any real habits for my asthma medicine but instead I take them when I remember to.	0.820	0.350	-0.014
Sometimes I test going without my asthma medicine to see what it’s like.	0.678	0.150	0.293
I don’t use my asthma medicine exactly like my doctor has said.	0.606	0.441	0.148
“self-adjusting the medication” Cronbach’s α = .919			
I don’t really follow the instructions for my asthma medicine but instead I can feel what my body needs. If I feel I need more, I take more. If I feel I need less, I take less.	0.260	0.889	0.038
I can tell myself how I feel and how much of my asthma medicine I need to take.	0.287	0.877	0.011
I don’t follow the doctor’s prescription of asthma medicine exactly but instead I can feel how much I need to take.	0.333	0.861	0.048
“concerns about side-effects” Cronbach’s α = 0.927			
I’m worried about how my asthma medicine will affect my body in the long run.	0.039	0.049	0.959
I worry about the side-effects of asthma medicine.	0.081	0.018	0.956

In order to demonstrate validity, responses to the developed questionnaire were compared with responses to the MARS to measure convergent and discriminant validity [23]. Responses to all items except the two covering concerns about the asthma medication correlated with the responses to the MARS (Table 5). The items in the three generated components were summarized and then measured against the MARS. The components called “medication routines” and “self-adjusting the medication” both correlated with the MARS. The component called “medication concerns” was not associated with the MARS (Table 5).

**Table 5:** Pearson’s correlation coefficients between the developed questionnaire and the MARS^a^ - phase 2

	Variables	MARS^a^
	Items	
1	It’s easy to forget asthma medicine.	-0.659^b^
1	I don’t have any real habits for my asthma medicine but instead I take them when I remember to.	-0.682^b^
1	I’m like a periodical drinker as far as my asthma medicine is concerned.	-0.649^b^
1	Sometimes I test going without my asthma medicine to see what it’s like.	-0.561^b^
1	I don’t use my asthma medicine exactly like my doctor has said.	-0.591^b^
2	I don’t really follow the instructions for my asthma medicine but instead I can feel what my body needs. If I feel I need more, I take more. If I feel I need less, I take less.	-0.596^b^
2	I can tell myself how I feel and how much of my asthma medicine I need to take.	-0.621^b^
2	I don’t follow the doctor’s prescription of asthma medicine exactly but instead I can feel how much I need to take.	-0.654^b^
3	I’m worried about how my asthma medicine will affect my body in the long run.	-0.094
3	I worry about the side-effects of asthma medicine.	-0.151
	**Scales**	
	medication routines	-0.762^b^
	self-adjusting the medication	-0.676^b^
	medication concerns	-0.126

^a^The Medication Adherence Report Scale

^b^Correlation is significant at the 0.01 level (2-tailed)

1 = item in the scale: medication routines

2 = item in the scale: self-adjusting the medication

3 = item in the scale: medication concerns

### Phase 3 - assessment of adherence in relation to personality traits and achieved goals

Descriptive statistics of personality traits and the developed adherence questionnaire are presented in Table 6. Neuroticism was positively associated with the scale called “medication routines” in the developed questionnaire (*r* = 0.321, *p* = 0.002), indicating that respondents scoring higher on this personality trait reported poorer adherence. Conscientiousness was negatively associated with the scale called “medication routines” (*r* = −0.313, *p* = 0.002) indicating that respondents scoring higher on this personality trait were more likely to be adherent to the prescribed asthma medication. No other associations were identified between the investigated personality traits and the developed adherence questionnaire.

**Table 6:** Means and standard deviations (SD) of personality traits and the developed adherence questionnaire (*n* = 104)

Personality traits	Mean (SD)
Neuroticism	27.57 (7.51)
Extraversion	43.45 (6.85)
Openness to experience	39.41 (5.50)
Agreeableness	46.97 (5.26)
Conscientiousness	46.71 (5.72)
Adherence	
Medication routines	11.11 (6.12)
Self-adjusting the medication	8.64 (4.34)
Medication concerns	4.07 (2.53)

In a multiple regression model (F 8.19, *p* = 0.001) explaining 13.6 % of the variance in the in adherence scale called “medication routines”, Neuroticism was identified as a positive predictor (B = 0.185, β = 0.242, *p* = 0.021) and Conscientiousness as a negative predictor (B = −2.44, β = −0.242, *p* = 0.021).

Regarding goals with the medication, 27 % of the respondents stated that they had not achieved their goal with the asthma medication. These respondents scored higher on the scale “medication routine” compared to respondents having achieved their goal (mean 13.63 SD 6.67 versus mean 10.00 SD 5.70), indicating poorer adherence. Six categories of goals with the asthma medication were reported: “To reduce the medication”, “To feel well”, “Symptom relief”, “Have a normal life”, “Enabling and facilitating physical activity” and “Long-term goals regarding the disease progress”.

## Discussion

The current study sought to develop a questionnaire with discriminatory power to measure adherence to asthma medication in adult individuals with asthma. An item pool was constructed that was based on our previous research and knowledge focusing adherence. Based on individuals with asthma randomly selected from the general population, who completed the initial questionnaire, ten items were selected and a revised version of the questionnaire was evaluated among another group of individuals with asthma also randomly selected from the general population. The final questionnaire contains three subscales- “medication routines”, “self-adjusting the medication” and “concerns about side-effects -, of which two correlated well with an established adherence questionnaire. The personality traits Neuroticism and Conscientiousness were associated with the subscale “medication routines”. Respondents who had not achieved their goals with the asthma medication reported poorer adherence.

The removal of several items that were regarded as having high/low endorsement was made to increase the possibility of discriminating the reported adherence. For instance, the MARS [25] has shown a skewed distribution in previous research [12, 14, 29], which could result in an overestimation of adherence. Consequently, the removal of the items with high/low endorsement was considered as a measure to enable a good response spread to facilitate the identification of high or low adherers, which could be viewed as an advantage with the developed questionnaire. The removal of the items could also be regarded as a limitation because it may affect both the respondents’ reporting on their beliefs and the distribution of their positions.

One strength is that the items in the final questionnaire were selected through analyses based on data derived from individuals with asthma selected at random from the general population. The focus of any questionnaire must be relevant to its target group [23], and participants in the current study consisted of individuals with asthma who had reported use of asthma medication, which could be a strength considering representativeness. However, as adherence to medication treatment tends to fluctuate over time, it may be a possible limitation that the current study did not provide data on the duration of the medication treatment. Moreover, the response-rate could have been higher, which could be seen as a shortcoming considering representativeness as it may be that those who chose to participate were those who were attentive to the medication treatment. Nevertheless, the sample sizes in both phase 1–3 (each with at least 100 participants) were considered sufficiently large enough. In addition, both samples were selected at random from a population-based study, which in turn consisted of participants selected at random, which could be regarded as a strength [27].

An additional strength of the current study may be that the questionnaire developed in phase 1 was tested in phase 2 and that the validity of the final questionnaire was tested in relation to a previously validated instrument, i.e. the MARS [25]. Responses to all items in the subscales “medication routines and “self-adjusting the medication” correlated with the responses to the MARS, which could be considered an appropriate validity check. It could be seen as a shortcoming that the developed questionnaire was not tested in relation to a more objective measure of adherence, for instance electronic monitoring of medication use. The scale “medication concerns” was not associated with the MARS, and thus removal of this subscale may be called for. However, concerns with asthma medication are to be regarded as one influencing factor of adherence [29].

Regarding personality and adherence, the current showed that the scale “medication routines” in the developed questionnaire – was associated with the personality traits Neuroticism and Conscientiousness. These findings are consistent with previous research focusing on personality traits and adherence to asthma medication [12–14] but also with adherence in relation to other long-term medication treatments [30–33]. Individuals who score higher on Neuroticism have reported poorer adherence to asthma medication treatment [14] and in relation to other long-term therapies [20, 33]. The personality trait Neuroticism measures degrees of emotional stability. Persons scoring higher on this trait could be more inclined to be worried, anxious, depressive and vulnerable to stress [15], which are characteristics that may explain the poorer adherence among individuals scoring higher on Neuroticism. Anxiety and depression, which are prevalent among individuals with asthma [34], have previously been associated with poorer adherence to asthma medication treatment [35]. For future studies, it would be advisable to include measures of anxiety and depression when assessing adherence to asthma medication treatment.

In contrast to Neuroticism, individuals scoring higher on Conscientiousness have reported higher adherence both in relation to asthma medication treatment [13, 14] and other medication treatments [30–33]. Importantly, the current study shows that individuals who had reached their goals with the asthma medication also reported better adherence, which indicates that adherence is of significance if personal goals with the asthma medication are to be achieved. Therefore, the current study suggests that individual goals with the asthma medication are discussed with the patients during follow-up consultations and that the set goals are used as inducement to promote adherence.

## Conclusions

A questionnaire consisting of three subscales - “medication routines”, “self-adjusting the medication” and “concerns about side-effects - of which two measure adherence behavior in relation to asthma medication and one measures concerns about the asthma medication– was developed and tested among individuals with asthma. The questionnaire appears to be useful for estimations of adherence to asthma medication in adult individuals with asthma but further testing is recommended. Two of the investigated personality traits – Neuroticism and Conscientiousness – were associated with one of the scales in the developed adherence questionnaire. Respondents who reported not having achieved their goal with the asthma medication reported poorer adherence.

## Abbreviations

Approx: Approximation
MARS: Medication adherence report scale
NEO-FFI: Neuroticism, extraversion and openness to experience five-factor inventory
SD: Standard Deviation
WSAS: West Sweden Asthma Study
